# Vascular injuries during gynecological laparoscopy – the vascular surgeon’s advice

**DOI:** 10.1590/S1516-31802005000100009

**Published:** 2005-01-02

**Authors:** Marcello Barbosa Barros, Francisco S. Lozano, Luis Queral

**Keywords:** Vascular injury, Laparoscopy, Gynecological surgery, Vascular Surgery, Lesão vascular, Laparoscopia, Cirurgia ginecológica, Cirurgia vascular

## Abstract

**CONTEXT:**

Iatrogenic vascular problems due to laparoscopy are a well recognized problem and lead to significant repercussions. In this context, a ten-year review of cases topic is presented, based on experience gained while heading two important vascular surgery services.

**CASES:**

Five patients with vascular injuries during elective laparoscopy are described. These patients presented with seven lesions of iliac vessels. All cases were evaluated immediately and required laparotomy, provisional hemostasis and urgent attendance by a vascular surgeon. Direct suturing was performed in three cases. One aortoiliac bypass and one ilioiliac reversed venous graft were made. Venous lesions were sutured. One case of a point-like perforation of the small bowel was found. There were no deaths and no complications during the postoperative period.

**DISCUSSION:**

Important points on this subject are made, and advice is given. There needs to be immediate recognition of the vascular injury, and expert repair by a vascular surgeon is recommended, in order to significantly reduce the degree of complications.

## RESUMO

**CONTEXTO:**

Iatrogenias vasculares nas laparoscopias são um problema bem reconhecido e podem levar a importantes repercussões. Neste contexto, são apresentadas importantes informações sobre o tema e descrição de casos durante a direção de dois importantes serviços de cirurgia vascular, abrangendo uma experiência de 10 anos.

**CASOS:**

São descritos cinco casos de lesão vascular durante laparoscopia eletiva, dentre os quais sete lesões de vasos ilíacos. Todos foram diagnosticados imediatamente e necessitaram de laparotomia, hemostasia provisória e intervenção de urgência por um cirurgião vascular. Em três casos foi realizada sutura direta. Realizou-se um *bypass* aorto-ilíaco e um enxerto ilíaco-ilíaco com safena invertida. As lesões venosas foram suturadas. Um caso de lesão puntiforme em intestino delgado foi encontrado. Não houve mortalidade ou complicações no periodo pós-operatório.

**DISCUSSÃO:**

Importantes considerações e conselhos a respeito do tema são apresentados. Deve haver reconhecimento imediato da lesão vascular e combinado ao reparo vascular especializado é recomendado para reduzir significativamente o grau de complicações.

## INTRODUCTION

Laparoscopy is a diagnostic and therapeutic technique used with increasing frequency by gynecologists, digestive tract surgeons, urologists and general surgeons. Generally, it is a safe and effective procedure, with associated morbidity of less than 4%,^1^ and it is well tolerated by patients. Laparoscopy has many advantages over conventional surgery, but it is not free of problems. Although infrequent, there have been reports of different iatrogenic lesions that may occur during laparoscopy. Vascular injuries are of great importance not only because they are responsible for problems of medical litigation,^2^ but also because of their significant morbidity and consequent mortality.^3-13^ In fact, they are the second most common cause of death during laparoscopy.^1^

Gynecological laparoscopy has certain particular characteristics: 1) a high number of indications (diagnostic, sterilization and therapeutic), and hence a greater theoretical possibility for complications; 2) it is carried out on the lower abdominal region, in a zone close to the iliac vessels; 3) it is often carried out on young women, and 4) it is generally carried out by professionals with little or no training in vascular repair.

With regard to iatrogenic vascular injuries and gynecological laparoscopy, there are many references in the literature in the form of studies in specific countries,^3-9^ reviews^3,5,10-12^ and case reports.^3-13^ However, only a few reports include the points of view of the vascular surgeons involved in such interventions.^10,11,13,14^ It was this latter aspect that prompted us to undertake the present study.

In this report, we review the experience of two vascular surgeons in relation to such injuries, with an analysis of cases that they have been involved in. Also, we make recommendations according to our point of view as vascular surgeons, so as to help in preventing these potentially life-threatening complications.

## CASES

The clinical records and operative reports of five patients who sustained injuries to the iliac vessels during elective laparoscopic surgery over a ten-year period were reviewed for this analysis. Each patient was analyzed with regard to age, anatomical location of the vascular injury, mechanism of injury, risk factors, blood loss, type of surgical repair, outcome and follow-up.

During the period between 1993 and 2003, five patients with seven iatrogenic iliac vessel injuries during gynecological therapeutic laparoscopic surgery were treated at the Union Memorial Hospital of Baltimore, Maryland, USA (three patients) and the University Hospital of Salamanca, Salamanca, Spain (two patients). All of the cases occurred during the first five years (1993-1997). Their mean age was 30.8 years (range: 23-42). The lesions were located in the left external iliac artery (one case), the right common iliac artery (two cases) and concomitantly in the right common iliac artery and vein (two cases). One patient also had a point-like perforation of the small bowel. The only lesion to the left vascular axis occurred during laparoscopic dissection in a patient with a history of previous laparoscopy (four years earlier) and total hysterectomy plus adnexectomy due to endometriosis and metropathic uterus (three years previously): this patient had intra-abdominal adherences. All lesions to the right vascular axis occurred when introducing the first trocar. One of the patients in particular was asthenic, while the others had no backgrounds or clinical data of interest (Table 1).

**Table 1:** Iliac arterial and venous injuries due to gynecological laparoscopy in five patients operated between 1993 and 2003 in two hospitals

Case	Hospital	Patient’s age	Risk factors	Location of iliac arterial lesion	Other lesions	Mechanism	Blood loss (liters)	Operation	Outcome	Follow-up*
1	UMH	27	None	Right common	None	Trocar/introduction	3.2	Suture	Survived	No sequelae
2	UMH	23	None	Right common	RCIV	Trocar/introduction	3.4	Suture	Survived	No sequelae
3	UMH	33	None	Right common	RCIV	Trocar/introduction	3.1	Suture	Survived	No sequelae
4	UHS	42	Adherences	Left external	None	Dissection	2.4	Venous graft	Survived	No sequelae
5	UHS	29	Thinness	Right common§	PIP†	Trocar/introduction	4.0	PTFE graft	Survived	No sequelae

*UMH = Union Memorial Hospital, Baltimore, Maryland, USA; UHS = University Hospital of Salamanca, Spain. RCIV = right common iliac vein; PIP = point-like intestinal perforation; PTFE = polytetrafluoroethylene.*

* *6-10 years.*

§ *At aortic bifurcation*

† *Suture.*

In all cases, immediate recognition of massive hemorrhaging made it necessary to perform laparotomy and manual compression of the abdominal aorta, and to call for an urgent intervention by a vascular surgeon. In three cases, direct suturing was performed; in another, an aortoiliac bypass was made (using polytetrafluoroethylene, PTFE); and in the last case, an ilioiliac reversed venous graft was interposed. The two venous lesions were sutured. The pointlike perforation of the small bowel was also sutured. The mean hemorrhage volume was 3.2 liters.

All patients survived and there were no complications during the postoperative period. The patients were discharged between the 9^th^ and 14^th^ days after the operation. None showed any resulting consequence during the follow-up period, which lasted six to ten years. None of the patients reported intermittent claudication, heaviness or limb edema. At the same time, noninvasive studies (duplex imaging) consistently revealed the permeability of the repairs made, with no images suggestive of pseudoaneurysm (Table 1). Nonetheless, two of the patients insisted on taking legal action.

## DISCUSSION

In addition to non-hospital causes of vascular lesions (trauma, violence, etc), intraoperative iatrogenic vascular injuries are an important cause for concern. Their diagnosis and treatment form part of the vascular surgeon’s duties. These kinds of iatrogenic injuries arise from conventional (open) surgery, endovascular techniques (angioplasty, stenting etc) and, naturally, endoscopy. In this sense, the surgeon faces different lesions, and different etiological agents and mechanisms are involved. In this light, we will focus on endoscopic lesions. These are considered to be important potential causes of morbidity and even death, if not treated appropriately and at the right time. In particular, gynecological laparoscopy is a field in which such complications are usually a cause for great concern and fear.

The incidence of vascular injuries during gynecological laparoscopy is fortunately very low. Studies in specific countries have reported an incidence of 0.1 cases per thousand procedures.^4,8^ This frequency is lower than what has been recorded for other lesions (intestinal, urethral or bladder).^7,9,15^ In addition, this percentage does not differ from what has been reported for open gynecological surgery^3,4^ or for general and digestive tract laparoscopy.^15-17^

Although it is uncommon even for vascular surgeons to have to treat such lesions, there needs to be an awareness of their existence, the problems involved, and the way in which they are best managed, since these are potentially life-threatening situations. In a review of the list of 408 trocar-related major vascular injuries notified to the Food and Drug Administration (FDA) by the medical device industry, between 1993 and the end of 1996, it was observed that 26 deaths occurred, thus representing a mortality rate of 6.37% for vascular laparoscopic injuries.^18^ A more recent report found a total of four deaths from 37 major vascular injuries of the aorta, vena cava and iliac vessels, thus representing a mortality rate of 10.81%.^19^

Failures in vascular repairs have also been described, which complicate the immediate postoperative period with acute ischemia or venous thrombosis. Ultimately, there may be arterial or venous consequences in the form of intermittent claudication or venous edema, in that order. Nonetheless, only a few publications^3,5,11,13^ provide information with respect to these extremes; our five patients did not suffer from postoperative complications or consequences, as assessed by duplex imaging after a mean follow-up period of ten years. Also, as in two of our cases, in such situations it is not uncommon for problems of medical litigation to occur, in which the vascular surgeon is also involved.^1,2,20^

The terminal aorta and iliac vessels are the most common sites for injuries, as reported in the literature. An associated between arterial and venous lesions, as seen in two cases in the present study, was found in 10% of the cases in the literature, overall.^5,10,12,21,22^

The mechanism responsible for the injury is intimately related to the laparoscopic technique and instruments used. The setup protocol (pneumoperitoneal needle or umbilical trocar) is the most common causative agent for vascular injury, as happened in our cases,^5-7,10-12,16,21^ although other mechanisms have been reported, relating to the operative procedure.^3,4,7,11,20^ Indeed, the most common mechanism is arterial or venous injury leading to rapidly recognizable hemorrhage, although later hemorrhaging cannot be ruled out. This would initially be contained by the pneumoperitoneum and the Trendelenburg position, or by a retroperitoneal hematoma, and may only become apparent hours after the laparoscopy.^4,11,22,23^ Finally, there have also been reports of vascular injuries that went unnoticed at the time they occurred and that were only discovered months after the laparoscopy, in the form of pseudoaneurysms^11^ or arteriovenous fistulas. We have not found any, but they do remain a possibility.

In the light of the above, nearly all publications have reported (and discussed) different guidelines and preventive measures, mainly focusing on those related to the actual laparoscopic technique.^1-13,16,23-29^ Despite this, the therapeutic aspects, especially in relation to vascular therapy, are not usually addressed in depth.^11,13,14^

Initially, the key therapeutic role is played by the gynecologist who performs the laparoscopy. The gynecologist must recognize any injury early on and carry out provisional, rapid and efficient hemostasis (manual, rather than with clamps). A review of the literature has highlighted this point since, if the diagnosis of the injury is made late, the mortality may reach up to 33%.^11^ In all our cases, these basic principles were fulfilled satisfactorily.

Although small hemorrhages can be controlled laparoscopically,^4,5,20,24^ injuries of the large vessels (aorta, vena cava or iliac vessels) require immediate performance of laparotomy to undertake proximal vascular control, because of the profuse bleeding that occurs. The fastest and best approach is a median laparotomy. The Pfannenstiel laparotomy, although practiced by many gynecologists, is not recommended because it hinders later vascular repair. One of our patients underwent this type of abdominal opening and this had to be converted to full median laparotomy.

Surgery of vascular traumas involves a method, tactics and technique that gynecologists are not usually familiar with. Apart from certain exceptional cases, it is best to call for a specialist in vascular surgery from the very start, after a hemorrhage has been detected; our cases are good examples of this. According to Chapron et al.,^5^ the vascular surgeon is not always consulted (71.4% of the cases).

The ideal situation is to perform a complete vascular repair, since it is counterproductive to ligate the common or external iliac arteries or veins. Usually, the arterial lesion will allow an arteriorrhaphy to be performed, using interrupted monofilament sutures so as to avoid stenosis.^3,10-13^ In lesions where this is not possible, because it would induce stenosis in the arterial lumen, the use of patch angioplasty (venous or synthetic) is indicated.^10,11,21^ On a few occasions, it is possible to section the artery, trim its edges, and perform anastomosis of the two ends.^11,21^ Finally, when the arterial lesion is very large and/or it is desired to perform an arterial resection (cases of intimal flap), the implantation of autologous or synthetic grafts is necessary to achieve arterial continuity, either by interposing the graft or in the form of a bypass.^3,10,11^ These were precisely the cases of our patients who required grafts.

In situations of contamination, the use of synthetic material should be avoided. Another technical detail that we consider important is the practice of arterial thrombectomy using a Fogarty probe, with cleaning of the arterial lumen using heparinized saline serum, in cases in which the manual hemostasis (together with instrumental clamping) has been very prolonged. In such situations, intravascular coagulation occurs distally to the clamp site, since such patients are not usually receiving anticoagulant therapy, as happens in conventional direct arterial surgery. Our two most severe cases required thrombectomy.

Wherever possible, venous ligation should be avoided, although it is referred to in some publications.^10,13^ It is better to repair a vein (venorrhaphy), even at the risk of subsequent thrombosis, than to ligate it. A ligated vein can never be recanalized and will therefore always lead to a greater degree of venous insufficiency. Sometimes, to expose an iliac vein appropriately, it is necessary to transect its homonymous artery first, and then repair both the vein and the artery. We used this technical maneuver in one of our patients.

Finally, two aspects of postoperative surveillance deserve comment: 1) the prevention of thrombosis in the iliac vein (even though this is not affected directly), since in this type of situation many specific thrombogenic factors are involved (contiguity to the trauma, hypovolemia etc) that are associated with other general factors typical of the postoperative period; and 2) the need for exhaustive followup over the short, medium and long-term, so as to rule out consequences arising from the surgery itself or unnoticed lesions.

In all cases, efficient interdisciplinary liaison between the gynecologist and the vascular surgeon is recommended, so that the severe problems that may arise from iatrogenic vascular injuries during gynecologic laparoscopy can be minimized. Although this is not generally the rule, the presence and help of a vascular surgeon, when dealing with an iatrogenic vascular injury, may often increase the chances of better solutions, since this can assist in managing the lesions in a more familiar and effective way.

## References

[1] Dixon M, Carrillo EH (1999). Iliac vascular injuries during elective laparoscopic surgery. Surg Endosc.

[2] Soutoul JH, Pierre F (1988). Les risques médico-légaux de la coelioscopie. Analyse de 32 dossiers de complicactions. [Medicolegal risks of celioscopy. Analysis of 32 cases of complications] J Gynecol Obstet Biol Reprod (Paris).

[3] Bergqvist D, Bergqvist A (1987). Vascular injuries during gynecologic surgery. Acta Obstet Gynecol Scand.

[4] Chapron C, Querleu D, Mage G (1992). Complications de la coeliochirurgie gynécologique. Etude multicentrique à partir de 7,604 coelioscopies. [Complications of gynecologic laparoscopy. Multicentric study of 7,604 laparoscopies]. J Gynecol Obstet Biol Reprod (Paris).

[5] Chapron CM, Pierre F, Lacroix S, Querleu D, Lansac J, Dubuisson JB (1997). Major vascular injuries during gynecologic laparoscopy. J Am Coll Surg.

[6] Marret H, Pierre F, Chapron C, Perrotin F, Body G, Lansac J (1997). Complications de la coelioscopie occasionnées par les trocards. Etude préliminaire dans le cadre du Registre national de la Société Française d’Endoscopie Gynécologique (SFEG). [Complications of laparoscopy caused by trocars. Preliminary study from the national registry of the French Society of Gynecologic Endoscopy]. J Gynecol Obstet Biol Reprod (Paris).

[7] Härkki-Sirén P, Kurki T (1997). A nationwide analysis of laparoscopic complications. Obstet Gynecol.

[8] Härkki-Sirén P, Sjöberg J, Mäkinen J (1997). Finnish national register of laparoscopic hysterectomies: a review and complications of 1165 operations. Am J Obstet Gynecol.

[9] Härkki-Sirén P, Sjöberg J, Kurki T (1999). Major complications of laparoscopy: a follow-up Finnish study. Obstet Gynecol.

[10] Baadsgaard SE, Bille S, Egeblad K (1989). Major vascular injury during gynecologic laparoscopy. Report of a case and review of published cases. Acta Obstet Gynecol Scand.

[11] Nordestgaard AG, Bodily KC, Osborne RW, Buttorff JD (1995). Major vascular injuries during laparoscopic procedures. Am J Surg.

[12] Geers J, Holden C (1996). Major vascular injury as a complication of laparoscopic surgery: a report of three cases and review of the literature. Am Surg.

[13] Witz M, Lehmann JM (1997). Major vascular injury during laparoscopy. Br J Surg.

[14] Nehler MR, Taylor LM, Porter JM (1998). Iatrogenic vascular trauma. Semin Vasc Surg.

[15] Munro MG (2002). Laparoscopic access: complications, technologies, and techniques. Curr Opin Obstet Gynecol.

[16] Saville LE, Woods MS (1995). Laparoscopy and major retroperitoneal vascular injuries (MRVI). Surg Endosc.

[17] Schäfer M, Lauper M, Krähenbühl L (2000). A nation’s experience of bleeding complications during laparoscopy. Am J Surg.

[18] Bhoyrul S, Vierra MA, Nezhat CR, Krummel TM, Way LW (2001). Trocar injuries in laparoscopic surgery. J Am Coll Surg.

[19] Sharp HT, Dodson MK, Draper ML, Watts DA, Doucette RC, Hurd WW (2002). Complications associated with optical-access laparoscopic trocars. Obstet Gynecol.

[20] Nezhat CR, Childers J, Borhan S (1996). Major Vessel Injury During Advanced Laparoscopic Surgery. J Am Assoc Gynecol Laparosc.

[21] Fruhwirth J, Lang PF (1997). Iatrogene Gefässläsionen bei laparoskopischen Eingriffen in der Gynäkologie. [Iatrogenic vascular lesions in laparoscopic interventions in gynecology]. Zentralbl Gynakol.

[22] Leron E, Piura B, Ohana E, Mazor M (1998). Delayed recognition of major vascular injury during laparoscopy. Eur J Obstet Gynecol Reprod Biol.

[23] Seidman DS, Nasserbakht F, Nezhat F, Nezhat C (1996). Delayed recognition of iliac artery injury during laparoscopic surgery. Surg Endosc.

[24] Charvolin JY, Querleu D, Lanvin D, Cosson M, Dubecq F (1997). Réparation coeliochirurgicale des complications opératoires de la coelioscopie en gynécologie. Expérience du pavillon Paul Gellé de Roubaix de 1992 à 1995. [Laparoscopic surgical repair of surgical complication of laparoscopy in gynecology. Experiences at the Paul Gelle de Rubaix Center from 1992 to 1995. J Gynecol Obstet Biol Reprod (Paris).

[25] Mirhashemi R, Harlow BL, Ginsburg ES, Signorello LB, Berkowitz R, Feldman S (1998). Predicting risk of complications with gynecologic laparoscopic surgery. Obstet Gynecol.

[26] Lin P, Grow DR (1999). Complications of laparoscopy. Strategies for prevention and cure. Obstet Gynecol Clin North Am.

[27] Bonjer HJ, Hazebroek EJ, Kazemier G, Giuffrida MC, Meijer WS, Lange JF (1997). Open versus closed establishment of pneumoperitoneum in laparoscopic surgery. Br J Surg.

[28] Woolcott R (1997). The safety of laparoscopy performed by direct trocar insertion and carbon dioxide insufflation under vision. Aust N Z J Obstet Gynaecol.

[29] Hanney RM, Carmalt HL, Merrett N, Tait N (1999). Vascular injuries during laparoscopy associated with the Hasson technique. J Am Coll Surg.

